# How much locomotive activity is needed for an active physical activity level: analysis of total step counts

**DOI:** 10.1186/1756-0500-4-512

**Published:** 2011-11-24

**Authors:** Kazunori Ohkawara, Kazuko Ishikawa-Takata, Jong Hoon Park, Izumi Tabata, Shigeho Tanaka

**Affiliations:** 1 Department of Health Promotion and Exercise, National Institute of Health and Nutrition, 1-23-1 Toyama, Shinjuku-ku, Tokyo 162-8636, Japan; 2 Faculty of Sport and Health Sciences, Ritsumeikan University, Shiga 525-8577, Japan

## Abstract

**Background:**

Although physical activity recommendations for public health have focused on locomotive activity such as walking and running, it is uncertain how much these activities contribute to overall physical activity level (PAL). The purpose of the present study was to determine the contribution of locomotive activity to PAL using total step counts measured in a calorimeter study.

**Methods:**

PAL, calculated as total energy expenditure divided by basal metabolic rate, was evaluated in 11 adult men using three different conditions for 24-hour human calorimeter measurements: a low-activity day (L-day) targeted at a low active level of PAL (1.45), and a high-frequency moderate activity day (M-day) or a high-frequency vigorous activity day (V-day) targeted at an active level of PAL (1.75). These subjects were permitted only light activities except prescribed activities. In a separate group of 41 adults, free-living PAL was evaluated using doubly-labeled water (DLW). In both experiments, step counts per day were also measured using an accelerometer.

**Results:**

In the human calorimeter study, PAL and step counts were 1.42 ± 0.10 and 8,973 ± 543 steps/d (L-day), 1.82 ± 0.14 and 29,588 ± 1,126 steps/d (M-day), and 1.74 ± 0.15 and 23,755 ± 1,038 steps/d (V-day), respectively. In the DLW study, PAL and step counts were 1.73 ± 0.15 and 10,022 ± 2,605 steps/d, and there was no significant relationship between PAL and daily step counts.

**Conclusions:**

These results indicate that an enormous number of steps are needed for an active level of PAL if individuals extend physical activity-induced energy expenditure by only locomotive activity. Therefore, non-locomotive activity such as household activity should also play a significant role in increasing PAL under free-living conditions.

## Background

The release of "Physical Activity and Public Health: A Recommendation from the Centers for Disease Control and Prevention and the American College of Sports Medicine" in 1995 spurred extensive discussion about the amount of physical activity (PA) needed to maintain good health [1]. More recently, the World Health Organization's (WHO's) "Global Recommendations on Physical Activity for Health" [2] following the 2008 "Physical Activity Guidelines for Americans" [3] has proposed more than 150 min of moderate-intensity PA per week to maintain body weight. The evidence for this recommendation was obtained from short-term clinical trials indicating that PA in the range of 13-26 metabolic equivalent (MET)-hours per week resulted in 1-3% weight loss, consistent with weight stability over the long term [4-6]. Thirteen MET-hours are roughly equivalent to brisk walking for 150 min.

In contrast, the PA recommendation for body weight management in the 2005 "Dietary Guidelines for Americans" [7] was adopted in large part from an Institute of Medicine (IOM) report [8]. These guidelines recommended approximately 60 min of above-moderate-intensity PA on most days of the week. This recommendation was primarily based on cross-sectional data on total daily energy expenditure (TEE) measured by the doubly-labeled water (DLW) method. Although differences in study design such as the use of clinical trials vs. cross-sectional studies likely contribute to the different PA recommendations, the use of different methods to measure PA may also play a role in these differences.

Physical activity-induced EE (PAEE) can be classified into two components: exercise-induced EE and non-exercise activity thermogenesis (NEAT) [9]. NEAT, a large component of daily PA, is the energy expended for everything that is not sleeping, eating, or sports-like exercise [10]. It includes the energy expended walking to work, performing yard work, undertaking agricultural tasks, and household activities such as typing, vacuuming, dishwashing, and fidgeting. Many of these activities can also be defined as non-locomotive activities. However, NEAT, especially NEAT due to non-locomotive activity, is difficult to measure under free-living conditions. In fact, only supervised exercise was counted towards PAEE in clinical trials that supported the WHO Global Recommendations for body weight management [4-6]. In contrast, in the IOM report, walking distance modeled for each activity level was estimated by a factorial approach to approximate TEE measured by the DLW method; any additional EE for achieving at the "active" level from the "sedentary" level was explained by brisk walking. We hypothesized that if non-locomotive activity was counted towards total PAEE, it could explain the discrepancy between the data used for the WHO Global Recommendations and that for the recommendations in the 2005 Dietary Guidelines for Americans. Moreover, this may also explain the discrepancy between walking equivalence as indicated in the IOM report and the average steps observed under free-living conditions. Therefore, the purpose of this study was to determine the contribution of locomotive activity to total PAEE based on the relationship between total step counts and PAL under free-living conditions and using a human calorimeter. This study results should also indicate a role of non-locomotive activity to increase PAL in a daily living.

## Methods

### Subjects

Subjects in the two protocols were recruited separately. The study protocols were approved by the Ethics Committee of the National Institute of Health & Nutrition, and signed informed consent was obtained from all subjects. Protocol 1: 11 adult men participated in a human calorimeter study. Age, height, weight, and body mass index (BMI) for subjects in Protocol 1 were 24.7 ± 5.8 year (mean, SD), 168.1 ± 3.9 cm, 64.5 ± 7.9 kg, and 22.8 ± 2.8 kg/m^2^, respectively. Protocol 2: Subjects were recruited through health care centers or at workplaces from various prefectures of the Kanto area (central Japan). 41 adults (12 males and 29 females) participated in a DLW study. Age, height, weight, and BMI for the subjects in Protocol 2 were 31.6 ± 9.1 year, 163.1 ± 8.9 cm, 57.8 ± 11.1 kg, and 21.6 ± 2.5 kg/m^2^, respectively. They were college students, housewives, or desk workers. They did not report care for aging parents but three of them engaged in care for their children. All subjects were free of chronic diseases that could affect metabolism or daily physical activity.

### Study concept

In Protocol 1 using a human calorimeter, each subject completed 24 h human calorimeter measurements under each of 3 different conditions. The concept of this study protocol was that subjects basically obtained PAEE from only prescribed locomotive activities since they were only permitted to carry out light activity in a sitting position during the rest of daytime. In Protocol 2 using DLW, subjects were measured total EE in a free-living condition. Obtained total EE should include PAEE induced by both of locomotive and non-locomotive activities. Thus, results from Protocol1 provide amount of locomotive activity for an active level of PAL if individuals extend PAEE from only locomotive activity. Furthermore, the gap of total step counts between Protocol 1 and 2 at same level of PAL may indicate the contribution of non-locomotive activity for maintaining an active level of PAL in daily-living condition.

### Human calorimeter (study 1 protocol)

In Protocol 1 using a human calorimeter, body weight and height were measured while subjects were in a fasting state. Each subject completed 24 h human calorimeter measurements under each of 3 different conditions: a low-activity day (L-day) targeted at a low active level of PAL (1.45), and a high-frequency moderate activity day (M-day) or a high-frequency vigorous activity day (V-day) targeted at an active level of PAL (1.75). The subjects went to bed at 2400 and were gently awakened at 0700 (7 h). After getting up, subjects were permitted to use the toilet and were required to return to bed immediately. Then, the subjects remained in a supine position without movement until 0800. Basal metabolic rate (BMR) was determined as the mean metabolic rate between 0715 and 0800. Coefficient of variation (CV) for BMR over 3 days was 1.7% as previously reported [11]. Prescribed physical activity in L-day consisted of 30 min of walking at 3.2 km/h, 30 min of walking at 5.6 km/h, and 15 min of jogging at 8.0 km/h. On the basis of the L-day, we modeled M-day and V-day targeted at 1.75 of PAL with additional walking or jogging time (Table 1). Except for prescribed activity including BMR measurement and eating, and use of the toilet, the subjects were only permitted to carry out light activity in a sitting position, such as reading, writing, and viewing television. Sleeping was not permitted during daytime. The order of the days was randomly determined for each subject. The experimental protocol was previously described in detail elsewhere [11].

**Table 1:** Amount of prescribed physical activity during 24-h calorimeter stays in Protocol 1 ^a^

	L-day	M-day	V-day
Normal walking (3.2 km/h)	30 min × 1	30 min × 1	30 min × 1

Brisk walking (5.6 km/h)	30 min × 1	30 min × 1	30 min × 1

		15 min × 11	15 min × 4

Jogging (8.0 km/h)	15 min × 1	15 min × 1	15 min × 4

Total	75 min	240 min	180 min

^a^L-day: low activity day, M-day: a day with high-frequency moderate physical activity, V-day: a day with high-frequency vigorous physical activity

An open-circuit indirect human calorimeter was used to measure 24-h EE and BMR [12,13]. Briefly, the respiratory chamber was an airtight room (20,000 L) equipped with a bed, desk, chair, TV with video deck, CD player, telephone, toilet, sink, and treadmill. The temperature and relative humidity in the room were controlled at 25°C and 55%, respectively. The oxygen and carbon dioxide concentrations of the air supply and exhaust were measured by mass spectrometry. For each experiment, the gas analyzer (ARCO-1000A-CH, Arco System, Kashiwa, Japan) was initially calibrated using a certified gas mixture and atmospheric air. The flow rate exhausted from the calorimeter was measured by pneumotachography (FLB1; Arco System, Kashiwa, Japan). The flow meter was calibrated before each measurement, and the flow rate was maintained at 90 L/min (ATP). *V˙ *O2 and carbon dioxide production (*V˙ *CO2) were determined by the flow rate of exhaust from the chamber, and the concentrations of the inlet and outlet air of the chamber, respectively [12]. EE was estimated from *V˙ *O2 and *V˙ *CO2 using Weir's equation [14]. The accuracy and precision of our human calorimeter for measurement of EE as determined by the alcohol combustion test was 99.8 ± 0.5% over 6 h and 99.4 ± 3.1% over 30 min. The subjects entered the chamber at 1750 and stayed until 1805 the next day. Sampling data were collected between 1800 and 1800 (24 h).

### Doubly-labeled water method (study 2 protocol)

Urine samples were collected early in the morning on the first study day at the study site, and body height and weight were also measured at that time. Then, a single dose of approximately 0.06 g/kg body weight of ^2^H_2_O (99.8 atom%, Cambridge Isotope Laboratories, MA, USA) and 1.4 g/kg body weight of H_2_^18^O (10.0 atom%, Taiyo Nippon Sanso, Tokyo, Japan) was administered orally to each subject. After isotope administration, participants were asked to collect urine samples on day 1 (the next day after the DLW dose) and on other 7 additional days (days 2, 3, 7, 8, 13, 14, and 15) during the study period at the same time of day in their home. On the last day, body weight was measured in the fasting state. Over the entire study days, the subjects were instructed to maintain their normal daily activities and eating patterns,

Gas samples for isotope ratio mass spectrometry (IRMS) were prepared by equilibration of urine samples with a gas. The gas used for equilibration of ^18^O was CO_2_, and H_2 _was used to equilibrate ^2^H. A Pt catalyst was used for equilibration of ^2^H. Urine was analyzed by IRMS using a DELTA Plus spectrometer (Thermo Electron Corporation, Bremen, Germany). ^2^H and ^18^O zero-time intercepts and elimination rates (k_H _and k_O_) were calculated using least-squares linear regression on the natural logarithm of the isotope concentration as a function of the time elapsed since dose administration. The zero-time intercepts were used to determine the isotope pool sizes. The TEE (kcal/day) calculation was performed using a modification of Weir's formula [14] based on the CO_2 _production rate (rCO_2_) and respiratory quotient (RQ). rCO_2 _was calculated as follows: rCO_2 _= 0.4554 × TBW × (1.007ko-1.041k_H_). The ratios of ^18^O and ^2^H dilution spaces were 1.030 ± 0.013 (Range; 1.001-1.056) and the coefficient of determination (R^2^) for multi-point regression equations was ≥ 0.99 for both ^18^O and ^2^H. These values were within recommended ranges by the International Atomic Energy Agency [15]. Food quotient (FQ) calculated by the equation of Black et al. was used instead of RQ [16]. The dietary survey for calculating FQ was conducted using a self-administered diet history questionnaire (DHQ) [17,18] which was reported on the validity of energy intakes [19]. In the present study, estimated average of FQ values were adopted in the groups of college students (FQ: 0.864), housewives (FQ: 0.872) and others (FQ: 0.880), respectively. This assumes that under conditions of perfect nutrient balance the FQ must equal the RQ [16,20].

### Basal metabolic rate and physical activity level

In Protocol 1, BMR was measured during human calorimeter stays, as further described in the "Human calorimeter" section. In Protocol 2, BMR was measured in the supine position in the early morning, 12 h or longer after the last meal, on the morning of the first or second visit to the study sites. The measurement was performed using a Douglas bag for 10 min × 2 with a 1 min break between measurements. After expired air was sampled, the O_2 _and CO_2 _concentrations were measured using a mass spectrometer (ARCO-1000, Arco System, Kashiwa, Japan) and the volume of expired air was measured with a certified dry gas meter (DC-5, Shinagawa, Tokyo, Japan). BMR was estimated from O_2 _consumption and CO_2 _production using Weir's equation [14]. CV for BMR over 3 days was 2.2% in this protocol. PAL was estimated by dividing TEE by BMR in both protocols.

### Anthropometry

A digital scale was used to measure body weight to the nearest 0.1 kg while subjects were dressed in light clothing. Barefoot standing height was measured to the nearest 0.1 cm using a wall-mounted stadiometer. Body mass index was calculated as body weight (kg) divided by height squared (m^2^).

### Step counts and physical activity energy expenditure

Step counts were measured using a uniaxial accelerometer (Lifecorder or Lifecorder EX, Suzuken Co. Ltd., Nagoya, Japan) in both protocols. Based on the previous study [21], PAEE in light, moderate, or vigorous intensity was also calculated in the DLW study. This accelerometer has been widely used in many countries due to its reasonable cost and reliable validity which could estimate EE for locomotive activity accurately [21-23]. The accelerometer was attached to the left side of the waist at the midline of the left thigh.

### Statistical analysis

All values are presented as means ± SDs. Differences were considered to be statistically significant if the *P *value was less than 0.05. In Protocol 1, the 24-h EE, BMR, PAL, and step count values obtained from the 3 conditions were compared by one-way analysis of variance with repeated measurements, and significant differences were analyzed using Scheffé's post-hoc test. In Protocol 2, correlations between step counts per day and PAL were assessed using Pearson's correlation coefficients (*r*). All statistical analyses were performed using SPSS version 14.0 J for WINDOWS (SPSS Inc, Chicago, IL).

## Results

**Figure 1:**
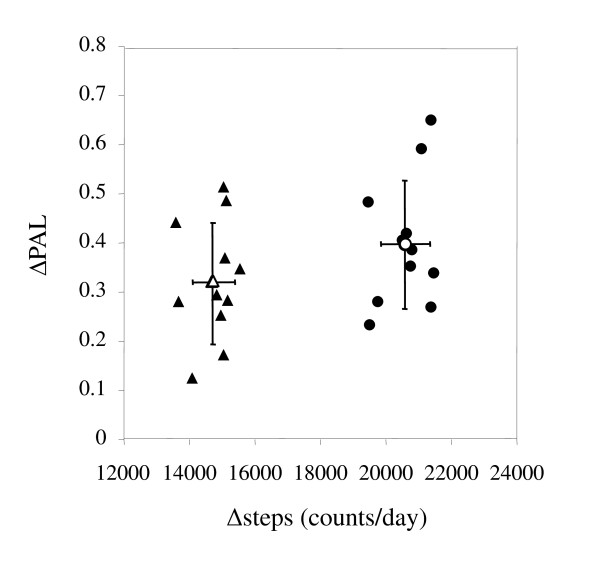
**Relationships between delta PAL and delta step counts calculated as M-day or V-day minus L-day in Protocol 1 using a human calorimeter**. Filled circles: values for a day with high-frequency moderate physical activity (M-day) minus values for a low active day (L-day), Filled triangles: values for a day with high-frequency vigorous physical activity (V-day) minus values for an L-day. Open circles or triangles and black lines are means ± SDs.

The results of Protocol 1 using a human calorimeter are shown in Table 2. There was no significant difference between BMRs on the L-day, M-day, or V-day. PALs on the M-day and V-day were significantly higher than on the L-day. According to the system of PAL categorization described in the IOM report [8], mean PAL values on the M-day and V-day would be classified as "active" and mean PAL on the L-day would be classified as "low activity". Figure 1 shows how many steps subjects would need to walk or jog throughout a day under controlled laboratory settings to increase PAEE from the "low activity" level to the "active" level. An additional 14,782 ± 650 steps/d corresponded to increase 0.32 ± 0.12 of PAL value calculated by subtracting L-day activity from V-day activity and an additional 20,615 ± 741 steps/d corresponded to a difference of 0.40 ± 0.13 PAL value between M-day and L-day activity.

**Table 2:** Energy expenditure, physical activity levels, and step counts during 24-h calorimeter stays in Protocol 1 ^a^

	L-day	M-day	V-day
24-h EE (kcal/day)	2228 ± 143	2816 ± 197^c^	2813 ± 163^c^

BMR (kcal/day)	1577 ± 129	1553 ± 114	1627 ± 157

Physical activity level ^b^	1.42 ± 0.10	1.82 ± 0.14^c^	1.74 ± 0.15^c^

Steps (counts/day)	8973 ± 543	29588 ± 1126^c^	23755 ± 1038^c d^

^a^Values are means ± SDs, L-day: low activity day, M-day: a day with high-frequency moderate physical activity, V-day: a day with high-frequency vigorous physical activity, 24-h EE: 24-hour total energy expenditure, BMR: basal metabolic rate

^b^Physical activity level was calculated as 24-h EE divided by BMR

^c^Significant differences compared with values for L-day in each variable (*P *< 0.05)

^d^Significant differences compared with values for M-day in each variable (*P *< 0.05)

**Figure 2:**
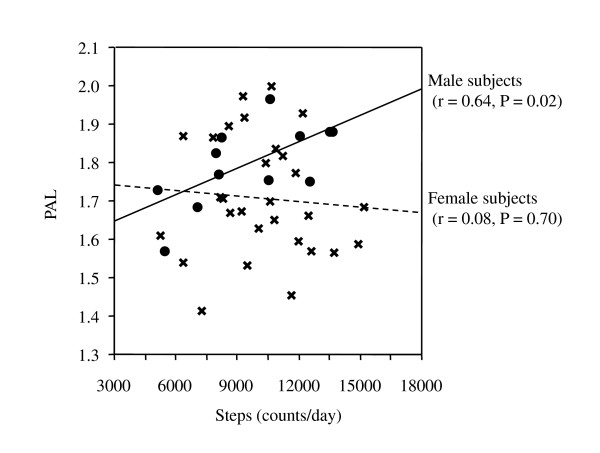
**Relationships between physical activity level (PAL) and step counts per day under free-living conditions in Protocol 2 using doubly labeled water**. Filled circles: male subjects, Crosses: female subjects.

Results from Protocol 2 using the DLW method are shown in Figure 2. Mean steps/d and PAL under free-living conditions were 10,022 ± 2,605 and 1.73 ± 0.15, respectively, among all subjects. The ranges of steps/d and PAL were 5,092-13,619 and 1.57-1.97, respectively, in male subjects (n = 12) and 5,288-15,242 and 1.41-2.00, respectively, in female subjects (n = 29). No significant relationship was observed between steps/d and PAL among all subjects (*r *= 0.06, *P *= 0.70) or in female subjects (*r *= 0.08, *P *= 0.70) although there was a significant relationship between these variables among male subjects (*r *= 0.64, *P *= 0.02). Furthermore, there was no significant relationship between PAEE in light, moderate, or vigorous intensity and PAL in either sex. Note that the step/d and PAL values in Protocol 2 are not necessarily representative values for healthy Japanese adults.

## Discussion

To clarify how much locomotive activity is needed for an active physical activity level, we examined the relationship between total step counts and PAL both under free-living conditions and in a human calorimeter. In the human calorimeter study, more than an additional 10,000 steps were needed to increase PAL from the "low activity" level (1.4-1.59 of PAL) to the "active" level (1.6-1.89 of PAL) as defined in an IOM report if PAEE was primarily due to walking or jogging (Table 3). On the other hand, in DLW study, average PAL and step counts under free-living conditions were 1.73 ± 0.15 and 10,022 ± 2,605 steps/d in 41 healthy adults. Thus, the gap of total step counts between these two study protocols was large even at a similar level of PAL. These results deduce that both of locomotive activity as well as non-locomotive activity such as typing, vacuuming, and dishwashing may be significant contributor to total PAEE in daily life.

**Table 3:** Physical activity level (PAL) categories and walking equivalence in the IOM report

PAL Category	PAL Range	PAL	**Walking Equivalence (km per day at 4.8-6.4 km per hour) **^**a**^		
			
			Lightweight Individuals (44 kg)	Medium-Weight Individuals (70 kg)	Heavy Individuals (120 kg)
Sedentary	1.0-1.39	1.25	0	0 (0 min)	0

Low activity	1.4-1.59				

Mean		1.5	4.6	3.5 (35 min)	2.4

Active	1.6-1.89				

Minimum		1.6	9.3	7.0	4.8

Mean		1.75	15.8	11.7 (125 min)	8.5

Very active	1.9-2.49				

Minimum		1.9	22.4	16.5	12.0

Mean		2.2	36.0	26.7 (285 min)	19.7

Maximum		2.	49.6	36.8	27.2

^a^In addition to energy spent for general activities in normal daily life

This table was modified from the table cited on page 161, chapter 5 in the 2005 Institute of Medicine (IOM) report

Even in human calorimeter studies, it is difficult to determine the relationship between PAEE from walking or jogging and step counts throughout a 24-hour protocol since subjects are typically permitted to engage *ad libitum *in light physical activity in addition to the prescribed exercises. de Jonge et al. [24] examined how much treadmill time is required to achieve 1.4 or 1.8 level of PAL in total 24-hour EE. The goal of that study was to develop a method for predicting an individual's 24-hour EE in a human calorimeter at these levels of PA. Average treadmill walking time was 177 ± 22 min on high-activity days (PAL: 1.78 ± 0.03) and 39 ± 9 min on low-activity days (PAL: 1.37 ± 0.02). These results indicate that 140 min of walking roughly corresponds to increase 0.40 of PAL. Note that subjects in that study conducted treadmill walking at 4.8 km per hour and at a 3% incline, and step counts were not reported in that study [24]. Subjects in the present study conducted all of their walking or jogging on a flat surface. Surprisingly, more than 120 min of extra walking time is needed to increase PAL from the "low activity" level to the "active" level, even if this PA consisted of combined brisk walking (5.6 km/h) and jogging (8.0 km/h) or walking at 4.8 km/h at a 3% incline. Furthermore, if we express PA in terms of step counts based on data from the present study, approximately 24,000 steps correspond to average 1.74 of PAL on a high-frequency vigorous-activity day and approximately 30,000 steps correspond to average 1.82 of PAL on a high-frequency moderate-activity day. The results of our DLW study found a maximum step count per day of approximately 15,000 steps, but the maximum PAL value was 2.00, classified as "very active" in the IOM report. Westerterp et al. [25] reported that the proportion of PAEE induced by standing, standing-active, and cycling was relatively large in 24-hour EE if subjects spent a normal day at 1.75 of PAL. Another study by Johannsen et al. [26] examined differences in posture allocation in daily living between lean and obese women using the Intelligent Device for Energy Expenditure and Activity (IDEEA) (MiniSun LLC, Fresno, CA), which analyzes the type, onset, duration and intensity of fundamental movements such as lying, sitting, standing, and locomotion. This study found that obese women spent significantly less time standing than lean women (163 ± 58 vs. 284 ± 134 min/day), although there was no significant difference in locomotive time between the two groups (lean: 60 ± 29 min/day, obese: 48 ± 16 min/day). Activities classified in the "standing" category included all non-locomotive activity. Thus, we can speculate that some people expend a large part of their PAEE due to non-locomotive activity.

A few previous studies have analyzed the relationship between step counts per day and PAL as measured by DLW under free-living conditions [27,28]. Fogelholm et al. [27] compared four different field measures of average daily EE with criterion data obtained by the DLW method in 20 overweight premenopausal women. In that study, the field measures (24-h activity measured by an accelerometer, reported vigorous activity, monitored vigorous activity as determined by heart rate, and daily steps) did not show a significant relationship with TEE adjusted for resting metabolic rate (*r *= -0.07-0.26; *P *> 0.20). In contrast, Colbert et al. [28] reported that in 56 older adults, a significant relationship was observed between daily step counts and PAEE adjusted for body weight determined using DLW (Spearman correlation coefficient = 0.585, *P *< 0.001). In the present study, a significant relationship between steps/d and PAL was observed only in male subjects. Thus, we speculate that women expend more energy through non-locomotive activity. Therefore, significant variability may be seen in the relationship between step counts per day and PAL in female subjects. However, variability was also observed in male subjects. This variability is likely due to several factors, such as culture, occupation, place of residence, age, etc.

Physical activity recommendations for body weight maintenance as well as chronic disease prevention have proposed conducting more than moderate intensity of physical activity [1-3]. On the other hand, the increase of PAL is strongly associated with body weight maintenance [7]. Our study results showed that there was a not significant relationship between total step counts and PAL in female subjects and the number of total step counts was relatively small in some subjects even though their PAL levels were around 1.75. Thus, light activities such as household activity may also contribute to obtain PAEE to reach at the recommended level. The acceptance of light activity for counting into total PA amount in the recommendations gives individuals some options of PA performance.

The strength point of this study was the use of human calorimeter and DLW method to measure 24-h energy expenditure accurately. However, there were some limitations in the present study: PA intensity such as light, moderate, or vigorous intensity should be considered for clarifying relationships between PAL and amount of PA. Additionally, we could not directly measure non-locomotive activity under free-living conditions. These were due to technical limitations. Furthermore, Only 11 males and 29 females participated in the DLW study, thus, future studies using a larger number of subjects are needed to further investigate the relationship between step count or PA amount in each intensity and PAL in both sexes, and to determine what factors are related to this variability.

## Conclusions

Our human calorimeter study showed that to increase PAEE from "low" to "active" levels through brisk walking, an additional 165 min of walking time at 5.6 km per hour (more than an additional 25,000 steps/d) was needed in subjects with a 65 kg body weight. These walking times and step counts were different from those determined under free-living conditions in our DLW study. These findings suggest that an enormous number of steps are needed for an active level of PAL if individuals extend physical activity-induced energy expenditure by only locomotive activity. Therefore, non-locomotive activity, a component of NEAT, may also play a significant role in increasing PAL under free-living conditions. Future studies are needed to clarify the contribution of non-locomotive activity to total PAEE using accurate measurement methods.

## Competing interests

The authors declare that they have no competing interests.

## Authors' contributions

KO, ST, and KI designed the study; KO, ST, KI, JP, and IT performed the experiments; KO, ST, and KI analyzed the data; KO, ST, and KI wrote a draft of the manuscript; JP and IT reviewed and edited the manuscript. All authors read and approved the final manuscript.

## References

[B1] Pate RR Pratt M Blair SN Haskell WL Macera CA Bouchard C Buchner D Ettinger W Heath GW King AC Physical activity and public health. A recommendation from the Centers for Disease Control and Prevention and the American College of Sports Medicine JAMA 1995 273 402 407 10.1001/jama.273.5.402 7823386

[B2] World Health Organization Global recommendations on physical activity for health 2010 26180873

[B3] Office of Disease Prevention & Health P US Department of Health and Human Services 2008 Physical Activity Guidelines for Americans 2008

[B4] Irwin ML Yasui Y Ulrich CM Bowen D Rudolph RE Schwartz RS Yukawa M Aiello E Potter JD McTiernan A Effect of exercise on total and intra-abdominal body fat in postmenopausal women: a randomized controlled trial JAMA 2003 289 323 330 10.1001/jama.289.3.323 12525233

[B5] McTiernan A Sorensen B Irwin ML Morgan A Yasui Y Rudolph RE Surawicz C Lampe JW Lampe PD Ayub K Potter JD Exercise effect on weight and body fat in men and women Obesity (Silver Spring) 2007 15 1496 1512 10.1038/oby.2007.178 17557987

[B6] Slentz CA Aiken LB Houmard JA Bales CW Johnson JL Tanner CJ Duscha BD Kraus WE Inactivity, exercise, and visceral fat. STRRIDE: a randomized, controlled study of exercise intensity and amount J Appl Physiol 2005 99 1613 1618 10.1152/japplphysiol.00124.2005 16002776

[B7] United States Dept. of Health and Human Services United States. Dietary Guidelines Advisory Committee: Dietary guidelines for Americans 2005 2005

[B8] Institute of Medicine Dietary Reference Intakes for Energy, Carbohydrate, Fiber, Fat, Fatty, Acids, Cholesterol, Protein and Amino Acids 2005 Washington, D.C.: The National Academies Press

[B9] Levine JA Eberhardt NL Jensen MD Role of nonexercise activity thermogenesis in resistance to fat gain in humans Science 1999 283 212 214 10.1126/science.283.5399.212 9880251

[B10] Levine JA Nonexercise activity thermogenesis (NEAT): environment and biology Am J Physiol Endocrinol Metab 2004 286 E675 685 10.1152/ajpendo.00562.2003 15102614

[B11] Ohkawara K Tanaka S Ishikawa-Takata K Tabata I Twenty-four-hour analysis of elevated energy expenditure after physical activity in a metabolic chamber: models of daily total energy expenditure Am J Clin Nutr 2008 87 1268 1276 18469249 10.1093/ajcn/87.5.1268

[B12] Futami J Tanaka S Yamamura C Oka J Ishikawa-Takata K Kashiwazaki H Measurement of energy expenditure by whole-body indirect human calorimeter--evaluation of validity and error factors J Jpn Soc Nutr Food Sci 2003 56 229 236 10.4327/jsnfs.56.229 14598913

[B13] Yamamura C Tanaka S Futami J Oka J Ishikawa-Takata K Kashiwazaki H Activity diary method for predicting energy expenditure as evaluated by a whole-body indirect human calorimeter J Nutr Sci Vitaminol 2003 49 262 269 10.3177/jnsv.49.262 14598913

[B14] Weir JB New methods for calculating metabolic rate with special reference to protein metabolism J Physiol 1949 109 1 9 15394301 10.1113/jphysiol.1949.sp004363 PMC1392602

[B15] Interneational Atomic Energy Agency Assessment of body composition and total energy expenditure in humans using stable isotope techniques 2009

[B16] Black AE Prentice AM Coward WA Use of food quotients to predict respiratory quotients for the doubly-labelled water method of measuring energy expenditure Hum Nutr Clin Nutr 1986 40 381 391 3771290

[B17] Sasaki S Yanagibori R Amano K Self-administered diet history questionnaire developed for health education: a relative validation of the test-version by comparison with 3-day diet record in women J Epidemiol 1998 8 203 215 10.2188/jea.8.203 9816812

[B18] Sasaki S Ushio F Amano K Morihara M Todoriki O Uehara Y Toyooka E Serum biomarker-based validation of a self-administered diet history questionnaire for Japanese subjects J Nutr Sci Vitaminol 2000 46 285 296 10.3177/jnsv.46.285 11227800

[B19] Okubo H Sasaki S Rafamantanantsoa HH Ishikawa-Takata K Okazaki H Tabata I Validation of self-reported energy intake by a self-administered diet history questionnaire using the doubly labeled water method in 140 Japanese adults Eur J Clin Nutr 2008 62 1343 1350 10.1038/sj.ejcn.1602858 17671444

[B20] Jones PJ Leitch CA Validation of doubly labeled water for measurement of caloric expenditure in collegiate swimmers J Appl Physiol 1993 74 2909 2914 8396112 10.1152/jappl.1993.74.6.2909

[B21] Kumahara H Schutz Y Ayabe M Yoshioka M Yoshitake Y Shindo M Ishii K Tanaka H The use of uniaxial accelerometry for the assessment of physical-activity-related energy expenditure: a validation study against whole-body indirect calorimetry Br J Nutr 2004 91 235 243 10.1079/BJN20031033 14756909

[B22] McClain JJ Craig CL Sisson SB Tudor-Locke C Comparison of Lifecorder EX and ActiGraph accelerometers under free-living conditions Appl Physiol Nutr Metab 2007 32 753 761 10.1139/H07-060 17622290

[B23] Schneider PL Crouter SE Bassett DR Pedometer measures of free-living physical activity: comparison of 13 models Med Sci Sports Exerc 2004 36 331 335 10.1249/01.MSS.0000113486.60548.E9 14767259

[B24] de Jonge L Nguyen T Smith SR Zachwieja JJ Roy HJ Bray GA Prediction of energy expenditure in a whole body indirect calorimeter at both low and high levels of physical activity Int J Obes Relat Metab Disord 2001 25 929 934 10.1038/sj.ijo.0801656 11443488

[B25] Westerterp KR Assessment of physical activity: a critical appraisal Eur J Appl Physiol 2009 105 823 828 10.1007/s00421-009-1000-2 19205725

[B26] Johannsen DL Welk GJ Sharp RL Flakoll PJ Differences in daily energy expenditure in lean and obese women: the role of posture allocation Obesity (Silver Spring) 2008 16 34 39 10.1038/oby.2007.15 18223609

[B27] Fogelholm M Hiilloskorpi H Laukkanen R Oja P Van Marken Lichtenbelt W Westerterp K Assessment of energy expenditure in overweight women Med Sci Sports Exerc 1998 30 1191 1197 10.1097/00005768-199808000-00002 9710856

[B28] Colbert LH Matthews CE Havighurst TC Kim K Schoeller DA Comparative Validity of Physical Activity Measures in Older Adults Med Sci Sports Exerc 2010 43 867 876 10.1249/MSS.0b013e3181fc7162 PMC3303696 20881882

